# Functional analysis of distraction arthroplasty in the treatment of ankle osteoarthritis

**DOI:** 10.1186/s13018-017-0519-x

**Published:** 2017-01-26

**Authors:** Hongmou Zhao, Wenqing Qu, Yi Li, Xiaojun Liang, Ning Ning, Yan Zhang, Dong Hu

**Affiliations:** 1 Foot and Ankle Surgery Department, Honghui Hospital, Xi’an Jiaotong University College of Medicine, 710054 Xi’an, China; 2 Department of Orthopaedic Surgery, Yantaishan Hospital, 264001 Yantai, China

**Keywords:** Ankle osteoarthritis, Arthroplasty, Distraction

## Abstract

**Background:**

Ankle joint distraction arthroplasty (AJDA) is an alternative surgical procedure for the management of moderate to severe ankle osteoarthritis. However, the benefit of this procedure and failure relative factors are still in debate. The purpose of the current study was to evaluate the functional outcomes of AJDA in treatment of moderate to severe ankle OA and to evaluate the relative factors correlated with treatment failure.

**Methods:**

Forty-six van Dijk stages II and III ankle osteoarthritis patients were included. Fifteen males and 31 females with a mean age of 54.8 (range, 42–71) years were followed with a mean of 42.8 (range, 24–68) months. The Ankle Osteoarthritis Scale (AOS) and American Orthopaedic Foot and Ankle Society (AOFAS) ankle-hindfoot score were used for functional outcome evaluation. The talar tilt (TT) angle and ankle joint space distance (AJSD) were evaluated. The risk ratio (RR) was calculated for each potential failure relative factor.

**Results:**

The AOS and AOFAS scores were significantly improved at the last follow-up time (*P* < 0.01). The AJSD was improved in 61% of patients and with a significant improvement compared with the preoperative conditions (*P* < 0.01). The TT angle and range of motion reached no significant difference. The failure rate was 21.7%. Patients with large TT (≥5°) angle (RR = 3.81, 95% CI 1.28–11.33, *P* = 0.02) and obesity (RR = 3.58, 95% CI 1.30–9.89, *P* = 0.01) were found to have positive correlation with failure. No correlation was found between failure and gender, or overweight, or side, or age, or type and stage of OA, or pin infection.

**Conclusions:**

The current study confirmed the early functional outcomes of ankle distraction arthroplasty. However, this procedure still has a relatively high failure rate, especially for those obese patients and patients with large TT angles.

**Electronic supplementary material:**

The online version of this article (doi:10.1186/s13018-017-0519-x) contains supplementary material, which is available to authorized users.

## Background

Osteoarthritis (OA) is a slowly progressing degenerative joint disorder that, in most cases, is diagnosed at a late stage after accompanying clinical symptoms. Ankle OA is one of the most common joint diseases and is a significant source of pain and disability for middle-aged and elderly people throughout the world [1]. It has many etiologies, and the posttraumatic OA that follows rotational ankle fractures or recurrent ligamentous instability is much more common [2, 3]. It is consensus on the joint-sacrificing procedures including total ankle replacement or ankle arthrodesis for the treatment of painful end-staged ankle OA [4–8]. However, all of these two procedures have downsides and are associated with limited long-term benefits [4, 5, 7]. And, patients with posttraumatic ankle OA are usually younger than patients with end-staged hip or knee OA [9]. Joint-preserving procedures may be a better treatment option than the joint-sacrificing procedures for those relatively younger patients and aim to relieve the symptoms, improve the quality of life, delay the progress of degeneration, and postpone the schedule of joint-sacrificing procedures [9, 10].

Ankle joint distraction arthroplasty (AJDA) is an alternative treatment method for moderate to severe ankle OA [10, 11]. This procedure was proved by basic and clinical researches that chondrocyte reparative activity may occur by unloading mechanical stresses [12–22]. However, some patients treated with AJDA reached unsatisfactory outcomes and were conversed to joint-sacrificing procedures [12, 17–22]. The reported failure rates were much different between studies [11, 12, 17–22]. van Valburg et al. [11] reported no failure (0/11) during a follow-up period between 10 and 60 months. However, Marijnissen et al. [17] reported a failure rate as high as 28% (13/46) during a similar follow-up period between 12 and 84 months. Prediction of failure on AJDA might be valuable as it will increase the clinical benefit and facilitate the clinical practice. The evidence-based literature that exists to support this procedure is still insufficient [9, 23], and the failure relative factors of this procedure are still in debate [12, 18, 20].

The authors hypothesized that AJDA was effective in treatment of ankle OA, and there might be some factors correlated with the failure of this procedure. The aim of the present study was to evaluate the clinical and radiological outcomes of AJDA in treatment of moderate to severe ankle OA and to evaluate the relative factors correlated with treatment failure.

## Methods

The current study was approved by the research board of our hospital. The authors retrospectively studied the clinical and radiological outcomes of joint distraction arthroplasty in treatment of ankle OA between June 2009 and June 2014. The inclusion criteria contained the following: (1) adults more than 18 years old, (2) symptomatic primary and posttraumatic ankle OA and failure of non-operative treatment more than 6 months, (3) with normal distal tibial articular alignment, (4) was treated with motional AJDA, and (5) with at least 24 months follow-up after the external fixator removal except treatment failure. The exclusion criteria contained the following: (1) combined with talar avascular necrosis or large cystoid variation that needed surgical intervention, such as osteochondral transplantation; (2) with distal tibial malalignment that needed realignment surgery; (3) old ankle or talus fracture malunion with the need for osteotomy or surgical reduction; (4) distraction time less than 10 weeks for any reason; and (5) patients with systemic diseases and comorbidities not suitable for AJDA.

Forty-six patients with 15 males and 31 females were included, and 20 have primary OA and 26 have traumatic OA. There were 22 left sides. The mean age at operation was 54.8 ± 6.3 (range, 42–71) years. The mean BMI was 24.3 ± 2.3 (range, 20.4–29.7) kg/m^2^. According to the van Dijk ankle OA classification [24], there were 27 stage II and 19 stage III.

### Surgical technique

**Fig. 1:**
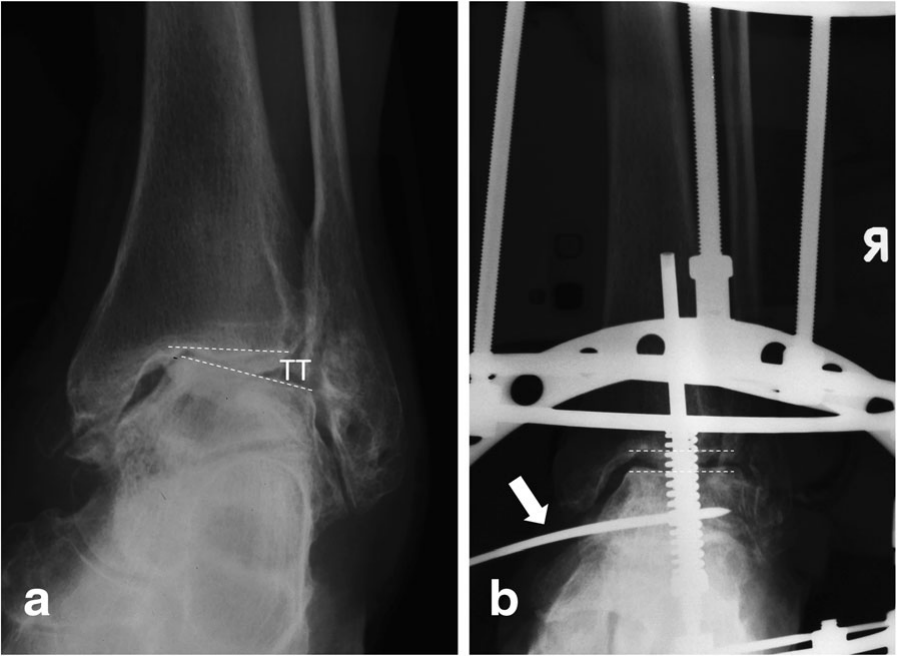
Anterior-posterior view of the ankle; the preoperative talar tilt (TT) angle was 12.2° (**a**), and a half pin was used from the medial to correct the tibiotalar joint parallel (**b**)

**Fig. 2:**
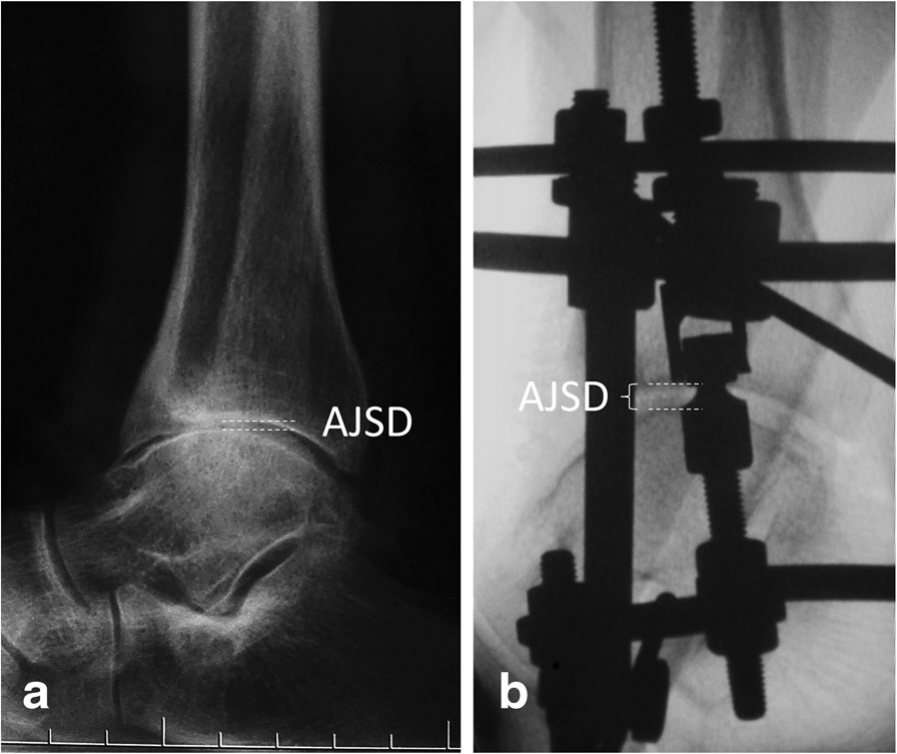
Lateral view of the ankle joint. The ankle joint space distance (AJSD) was distracted from 1.7 mm (**a**) preoperatively to 5.2 mm (**b**) postoperatively

All of the included patients were treated with open debridement and Ilizarov AJDA. The surgical technique has been well described in the literatures [11, 20, 21, 25]. Before the external fixator was applied, medial-anterior or lateral-anterior or combined incisions were used, according to the preoperative radiological evaluations, to clear the osteophytes and spurs. The cartilage debridement or microfracture is performed if the patient has a cartilage lesion. Also, the inflammatory synovial tissue was cleared. A drainage tube was placed after washing, and then, the layer is sutured and the incision is closed. Immediately following open operation, the Ilizarov external ring fixator was applied. If the patient has preoperative increased talar tilt (TT) angle, a half pin from the medial side is drilled to the talus for distraction (Fig. 1). This pin was connected to the calcaneal ring with a rod. The ankle joint space distance (AJSD) was distracted 5 mm during operation as measured with the use of fluoroscopy (Fig. 2). The ankle was placed through a range of motion under fluoroscopy to check the amount of distraction as well as to double check the alignment. Sterile dressings were placed on the wounds. Full weight-bearing was allowed 2 weeks postoperation. The external fixator was removed 12 weeks in average (range, 10–14 weeks).

### Clinical and radiographic examination

The radiological evaluation contained the TT angle in weight-bearing anterior-posterior ankle X-ray (Fig. 1a). The AJSD was measured in weight-bearing lateral X-ray (Fig. 2). The American Orthopaedic Foot and Ankle Society (AOFAS) ankle-hindfoot score [26] and the Ankle Osteoarthritis Scale (AOS) were used preoperatively and at the follow-up time to evaluate the functional outcomes [27]. During the follow-up time, the patient with increased AOS total score, or with increased symptoms that need revision, including open or arthroscopic debridement, supramalleolar osteotomy, ankle arthroplasty, or arthrodesis, was defined as treatment failure. The functional and radiological outcomes of those failure patients before further surgical intervention were included as the last follow-up conditions. The BMI cut-off values of 24 and 28 kg/m^2^ for overweight and obesity, respectively, recommended for the Asian population were used in the current study [28].

### Statistical analysis

Descriptive statistics were calculated as mean ± standard deviation. Statistical analysis of the included data was performed using Student’s *t* test with the level of significance set at α = 0.05. According to the failure rate, the risk ratio (RR) and 95% confidence interval (CI) were calculated for each potential relative factor. The statistical analyses were performed with the SPSS 17.0 software (SPSS Inc., Chicago, IL).

## Results

The average distraction time was 12.2 ± 1.0 (range, 10–14) weeks. A total of 14 cases with 27 pin infections were found; all of them were treated with local dressing and oral or intravenous antibiotics, and no one needed early removal of external fixator because of pin infection. No other complications such as fracture or major nerve and blood vessel injury were found.

**Fig. 3:**
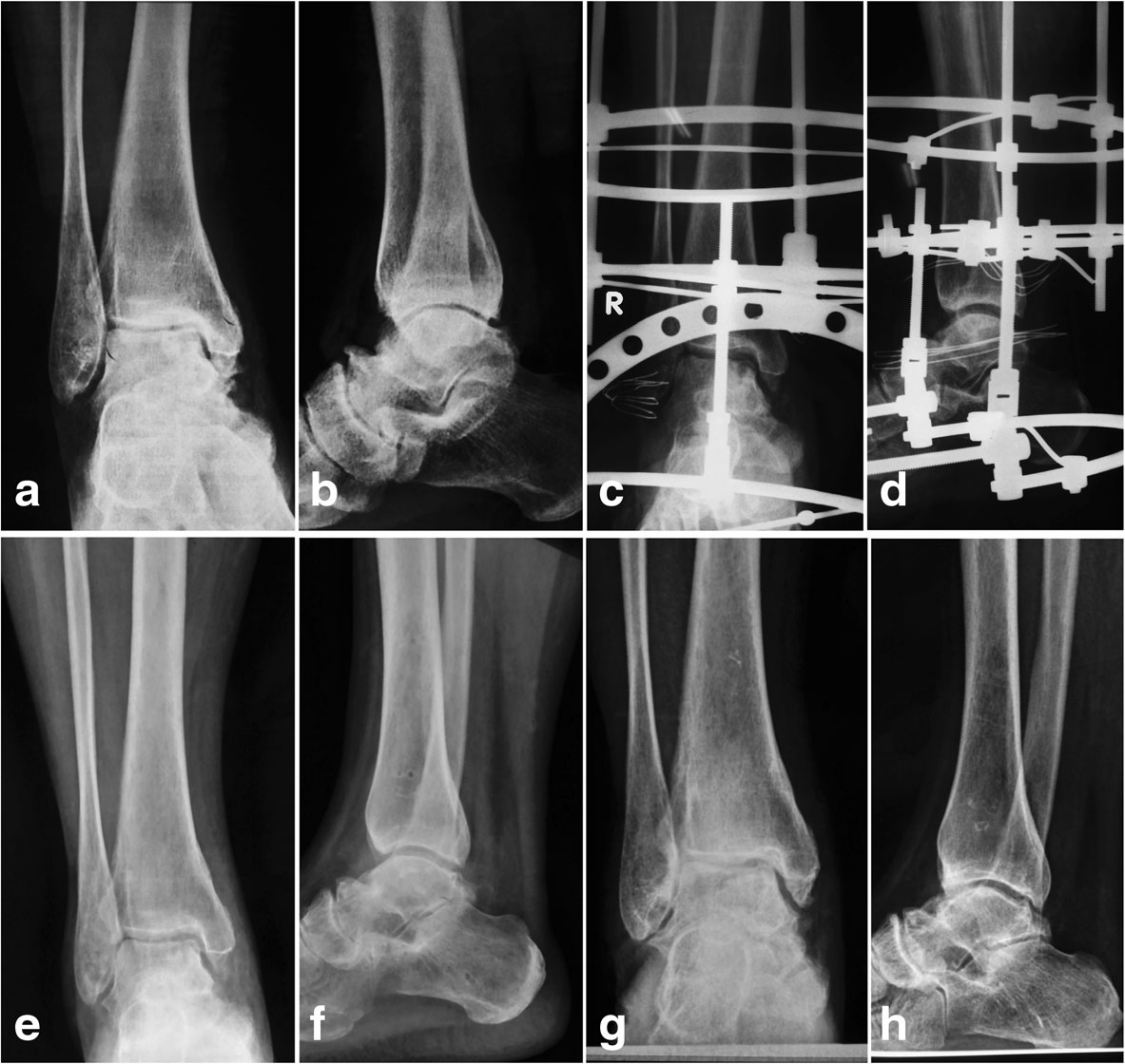
Preoperative X-ray of a 51-year-old female showed a van Dijk stage II osteoarthritis of the right ankle joint; the preoperative ankle joint space distance was 2.3 mm (**a**, **b**). The patient was treated with open debridement and distraction arthroplasty; the postoperative X-ray showed clear joint space and was enlarged to 6.0 mm (**c**, **d**). The 1-year follow-up X-ray showed clear joint space and diminishing of subchondral sclerosis, and the joint space was 4.6 mm (**e**, **f**). The 3-year follow-up X-ray showed that the joint space is still clear and with a distance of 3.8 mm (**g**, **h**), and the patient was pain-free

The mean follow-up time was 42.8 ± 10.2 (range, 24–68) months after the external fixator removal. The mean AOFAS ankle-hindfoot score and AOS pain and functional scores were all improved significantly (*P* < 0.01) (Table 1). The AJSD was significantly improved with a mean of 1.0 ± 0.6 (range, 0–2.3) mm. In comparing with the preoperative condition, the AJSDs were improved in 28 (61%) cases and maintained at the last follow-up time (Fig. 3). The mean TT angle was decreased from 3.8° preoperatively to 3.1° at final follow-up; however, with the numbers available, no significant difference could be detected (*P* = 0.06). Also, the ROM of ankle joint reached no significant improvement (*P* = 0.27).

**Table 1:** The functional outcomes of preoperative and last follow-up time (*n* = 46)

	Preoperative	Last follow-up	*P* value
AOS total	5.26 ± 0.51	4.19 ± 0.91	0.00
AOS pain	4.39 ± 0.33	3.53 ± 0.89	0.00
AOS function	6.13 ± 0.70	4.85 ± 0.96	0.00
AOFAS	63.5 ± 5.8	73.8 ± 8.6	0.00
AJSD	2.2 ± 0.8	3.1 ± 0.7	0.00
TT	3.8 ± 2.0	3.1 ± 1.5	0.06
ROM	38.2 ± 6.5	39.7 ± 6.4	0.27

*AOS* Ankle Osteoarthritis Scale, *AOFAS* American Orthopaedic Foot and Ankle Society ankle-hindfoot scale, *TT* talar tilt angle, *AJSD* ankle joint space distance, *ROM* range of motion of the ankle joint

During the follow-up time, 10 patients (21.7%) were diagnosed as failure between 6 and 42 months after frame removal. Five patients were failure within 1 year, 3 in the second year, and 2 in the third and fourth year, respectively. Six of them were treated with ankle fusion; the other 4 patients were treated with debridement, injection, and extracorporeal shock wave therapy, and still in the schedule of arthrodesis. According to the failure rate, the RR and 95% CI of potential relative factors were calculated and found that the preoperative large TT angle (≥5° [29]) and obesity were positively correlated with high failure rate (Table 2). No correlation was found between failure and gender, or overweight, or side, or age, or type and stage of OA, or pin infection. Also, the improvement of AOFAS score was significantly smaller in the patients with preoperative large TT angle (*P* = 0.02) and obesity (*P* = 0.01) (Table 3).

**Table 2:** The risk relative factor analysis of treatment failure (*n* = 46)

Relative factor	Failure/sample	Risk ratio	95% CI	*P* value
TT ≥ 5°	7/13	3.81	1.28–11.33	0.02
BMI ≥ 24 kg/m^2^	8/27	2.81	0.67–11.81	0.16
BMI ≥ 28 kg/m^2^	2/3	3.58	1.30–9.89	0.01
Age ≥ 55 years	6/19	2.13	0.69–6.54	0.19
Female	8/31	1.94	0.47–8.02	0.36
Left	5/22	1.09	0.36–3.27	0.88
van Dijk stage III	6/19	2.13	0.69–6.54	0.19
TOA	5/26	1.30	0.44–3.88	0.64
Pin infection	5/14	2.21	0.76–6.44	0.14

*CI* confidence interval, *TT* talar tilt angle, *BMI* body mass index, *TOA* traumatic osteoarthritis

**Table 3:** The effect of relative factors on the functional outcomes (*n* = 46)

Relative factor	Cases of study/control	Improvement of AOFAS score		*P* value
		Study	Control	
TT ≥ 5°	13/33	4.8 ± 11.1	12.0 ± 8.2	0.02
BMI ≥ 24 kg/m^2^	27/19	8.4 ± 9.7	13.2 ± 9.5	0.10
BMI ≥ 28 kg/m^2^	3/43	−2.7 ± 4.0	11.3 ± 8.9	0.01
Age ≥ 55 years	19/27	7.8 ± 11.7	12.1 ± 7.1	0.13
Female	31/15	9.9 ± 10.0	11.2 ± 7.8	0.66
Left	22/24	10.5 ± 9.3	10.2 ± 9.5	0.91
van Dijk stage III	19/27	8.4 ± 9.7	11.8 ± 8.8	0.22
TOA	26/20	9.6 ± 10.0	11.5 ± 8.3	0.50
Pin infection	14/32	8.3 ± 10.6	11.3 ± 8.7	0.32

*AOFAS* American Orthopaedic Foot and Ankle Society ankle-hindfoot scale, *TT* talar tilt angle, *BMI* body mass index, *TOA* traumatic osteoarthritis

## Discussion

Joint distraction arthroplasty with external fixation is a low-risk procedure that offers a promising solution to a complex problem [21] and has evolved as an alternative treatment to joint-sacrificing procedures like ankle arthrodesis or replacement [9, 10, 13]. This procedure was proved to improve the cartilage proteoglycan metabolism, decrease the inflammation reaction, repair of cartilage atrophy, and promote repair of osteochondral defect in the weight-bearing area from basic researches [14–16]. The AJDA was firstly introduced by French authors Judet R and Judet T in 1978 [30] and popularized after van Valburg’s report in English in 1995 [11]. In 1996, Buckwalter [31] commented that van Valburg and colleagues’ report added a potentially important new approach to the treatment of ankle OA, especially for young active people, and called for long-term studies with objective parameters to prove whether joint distraction could be the treatment of choice for ankle OA. In 1999, the first prospective data became available with a 2-year follow-up [22]. And till now, nine clinical studies about AJDA in English were searched from the database (Additional file 1: Table S1) [11, 12, 17–22, 32]. The results of these studies showed that AJDA could effectively decrease the pain [11, 17, 19–22, 32], improve the functional outcomes [16, 18–20, 31], enlarge the joint space width [11, 17], and decrease the subchondral bone density [17, 22, 32]. However, there are still with controversy and the evidence with limitations. Therefore, we clinically and radiographically followed up our 46 patients with moderate to severe ankle OA treated with AJDA to provide the further evidence for this procedure. From the results of present study, AJDA showed significant improvement of pain and functional outcomes, even included the scores of those patients diagnosed as failure at the last follow-up time before further surgical intervention (Table 1).

Two main distraction methods were reported in the literature. The first one was gradual distraction, 0.5 mm twice daily for 5 days to get an over distraction of 5 mm, and starting the day after application of the apparatus [11, 17, 19, 22]; the other method, used in our patients, was distracted approximately 5 mm acutely in the operation room [18, 20, 21, 32]. For the second method, the physicians can confirm the AJSD during the operation to make sure that an enough distraction was done and to check and correct the hinges along Inman’s axis during dorsiflexion and plantar flexion of the ankle joint after distraction, to prevent uneven joint distraction through a range of motion [21]. The final AJDS after distraction should be more than 5 mm because it will decrease after weight-bearing. Fragomen et al. [33] recommended at least 5.8 mm AJSD in distraction to ensure no contact between joint surfaces according to a biomechanical study. Sometimes, we added gradual distraction during the short hospital stay after operation.

According to current results, the AJSD was enlarged from mean 2.2 mm preoperatively to 3.1 mm at the last follow-up time, with an improvement of 41% and maintained in 61% (28/46) of cases. This was in accordance with the previous reports [11, 17]. We did not found significant improvement in mobility of the ankle joint after distraction, and for sure, increase of mobility is not the primary aim of AJDA. The authors agree with Pagenstert et al. [34] that for the treatment of ankle OA, the improvement of pain correlated with walking ability and general activity but did not correlate with achieved ROM.

In the current study, 13 patients with increased TT angle (≥5° [29]) were included. A half pin from the medial side was drilled to the talus for distraction, and the TT was corrected to normal during operation and conformed by fluoroscopy (Fig. 1). After the extra pin in the talus was used, the hindfoot was forced to the valgus, the medial structure was distracted, and the lateral structure was relaxed. It was expected that after 3 months distraction, the medial and lateral soft tissue recovery, in some degree, might contain in some cases. However, the postoperative TT was not well corrected in most of the cases with a failure rate as high as 54% (7/13) in these patients. No study was published focusing on the correction of TT angle with AJDA. However, increased TT angle is not a rare condition in ankle OA patients although without etiological data. In the studies focused on the supramalleolar osteotomy in treatment of the varus ankle OA, the percentage of patients with increased TT angle was as high as 44 to 61% [29, 35]. From current results, in the ankle OA patients with incongruent joint relationship, restoring the joint congruence and normal weight-bearing alignment might be more important than enlarging the decreased joint space. AJDA combined with lateral collateral ligaments reconstruction, medial release, and realignment osteotomy may be helpful in these cases, but the clinical evidence is still needed.

Six of the nine clinical studies focused on AJDA included 168 cases that reported the failure number and time [11, 17–19, 22, 32], the combined failure rate of 23.2% in a mean follow-up time of 51.8 months. Current results showed a failure rate of 21.7% with a mean follow-up time of 43 months. The failure rate of AJDA is higher compared with that of total ankle replacement or ankle arthrodesis. The systematic review of Zhao et al. [7] showed the failure rate of STAR total ankle replacement and reported a pooled 5-year survival rate of 85.9%, and the pooled 10-year survival rate was 71.1%. Kim et al. [36] gave a meta-analysis and reported the risk of re-operation, and major surgical complications were significantly increased in the total ankle replacement group while compared with ankle arthrodesis, which means that ankle arthrodesis have an even smaller failure rate. But, the authors agree with Nguyen et al. [37] that the ankle function after joint distraction declines over time; however, this does not compromise any future arthroplasty or arthrodesis, if required.

For these 39 failure patients in the included literatures [11, 17–19, 22, 32], there were 20 within the first year postoperation, 6 in the second year, 6 between 3 and 5 years, and 7 between 6 and 17 years. This might implied that most of the failure of AJDA for ankle OA occurred within the first 5 years (82%) and especially within the first year (51%) [11, 17–19, 22, 32]. The decline of failure rate with time gave us a clue that some potential relative factors might play roles in this procedure. Nguyen et al. [18] reported that the positive predictors of ankle survival included a better AOS score in 2 years postoperation (*P* = 0.04) and older age at surgery (*P* = 0.04) and fixed distraction (*P* < 0.01). However, Saltzman et al. [20] reported that motion distraction group had significantly better AOS scores than the fixed distraction group at each time point after frame removal (*P* < 0.01). It was interesting that the totally opposite results were concluded from the same patients with different follow-up time [18, 20]. Marijnissen et al. [12] reported that the survival analysis showed positive correlation between failure and gender and with a higher percentage failure in women (*P* < 0.01). According to current study, the RR analysis showed that the obesity (BMI ≥ 28 kg/m^2^) and large TT angle (≥5° [28]) were positively correlated with failure. The relationship between BMI and failure was evaluated in a previous study but with a negative result (*P* = 0.41) [12]. This inconsistence might be due to the ethnic differences. For those patients with large preoperative TT angle, single distraction could not effectively realign the weight-bearing line of the ankle and hindfoot according to current results. Supramalleolar or calcaneal osteotomy may be helpful in this condition according to previous studies [35, 38]. We did not found the positive correlation between failure and age; this confirmed the results by Marijnissen et al. [12]. Also, no positive correlation was found between failure and gender, or overweight, or side, or type and stage of OA, or pin infection.

The limitations of the current study include the limited duration of follow-up, the retrospective design and the lack of control group and information on the intra-articular. Although the outcomes will change by time, our early results confirmed that the functional outcomes of AJDA are good in terms of pain relief and enlargement of ankle joint space. Most of the failures happened within the first 2 years (66.7%) according to previous studies [11, 17–19, 22, 32]. All of our included patients had a minimum follow-up of 2 years or were considered failure within 2 years after distraction, with a mean follow-up time of 42.8 months, and it is enough to evaluate the failure rate and relative factors. Because of the relatively small sample size of current study, most RR values of potential failure relative factors were with large 95% CI. But, we still could find the strong positive correlation between treatment failure and obesity, and large TT angle. According to current results, the well-designed large sample and long-term follow-up prospective studies that focused on the AJDA are still needed.

## Conclusions

In conclusion, distraction arthroplasty can effectively improve the functional outcomes of patients with moderate to severe ankle OA, and the joint space enlarged significantly. Also, this procedure might delay the need for joint-sacrificing operations in some patients. However, the joint distraction arthroplasty should be cautiously used in obese patients and those patients with larger talar tilt angle because of high failure rate.

## Additional file

Additional file 1: Table S1. Clinical outcomes of distraction arthroplasty in treatment of ankle osteoarthritis from literature. (DOCX 25 kb)

## Abbreviations

AJDA: Ankle joint distraction arthroplasty
AJSD: Ankle joint space distance
AOFAS: American Orthopaedic Foot and Ankle Society
AOS: Ankle Osteoarthritis Scale
BMI: Body mass index
CI: Confidence interval
OA: Osteoarthritis
ROM: Range of motion
RR: Risk ratio
TT: Talar tilt
