# Supplementary material for: Functional analysis of distraction arthroplasty in the treatment of ankle osteoarthritis
Source: J Orthop Surg Res. 2017 Jan 26;12:18. doi: 10.1186/s13018-017-0519-x (PMC5270240; doi:10.1186/s13018-017-0519-x)
Supplement: Additional file 1: Table S1. — Clinical outcomes of distraction arthroplasty in treatment of ankle osteoarthritis from literature. (DOCX 25 kb) [file 13018_2017_519_MOESM1_ESM.docx]

**Table S1.** Clinical outcomes of distraction arthroplasty in treatment of ankle osteoarthritis from literature.

| Author/year | Sample with FU | Methods | Evaluation | FU (mth) | Outcomes | Conclusions | Failure and times |
| --- | --- | --- | --- | --- | --- | --- | --- |
| van Valburg 1995 [11] | 11 | Retrospective; 0.5mm twice daily to 5mm in total; 8 (6-12) wk fixed distraction, and 15 (12-22) wk in total. | pain, ROM, JSW, joint hydrostatic pressure | 20 (10-60) | Pain decreased in all patients; ROM improved in 55%; joint space widening in 50%; joint hydrostatic pressure increased during distraction. | AJDA may delay the need for ankle arthrodesis as a result of clinical improvement. | no |
| van Valburg 1999 [22] | 17 | Prospective; same to van Valburg [11]; 3 mth fixed distraction. | pain, function, ROM, bone density | 24 | Physical and functional impairment, and pain were all improved (*P*＜0.05); 75% increase in mobility; all cases with diminished subchondral sclerosis. | AJDA is a promising treatment for severe ankle OA, at least delaying the need for a joint fusion. | 4 failures within 1 yr |
| Marijnissen 2002 [17]* | 46 | Prospective; same to van Valburg [11]; 3 mth fixed distraction. | pain, function, JSW, ROM, bone density | 37 (12-84) | Pain score, function score, JSW, bone density were all improved (*P*＜0.05), the improvement increased over time. ROM reached NSD. | AJDA has significant clinical benefit in treatment of severe ankle OA. | 13 failures; 8 within 1 yr; 3 in 2 yr; 1 in 3 yr and 1 in 4 yr |
| Marijnissen 2002 [17]* | 17 | RCT; distractions versus debridements; same to van Valburg [11]; 3 mth fixed distraction. | pain, function, JSW, ROM, bone density | 12 | Pain, function and clinical status improved better in distraction group (*P*＜0.05); Changes of JSW and subchondral sclerosis reached NSD. | AJDA showed better improvement in functional outcomes than debridement alone. | 3 failures in debridement group |
| Ploegmakers 2005 [19] | 22 | Retrospective; same to van Valburg [11]; 15 (12-22) wk fixed distraction. | Pain, function, clinical status, AOS | 120 (84-168) | Pain, function and clinical status improved (*P*＜0.01); ROM increased by 34±23%, but NSD; AOS pain, disability, and total scores were all improved (*P*＜0.01). | AJDA for treatment of severe ankle OA is followed by significant clinical benefit for a period of time. | 6 failures; 5 fusion; 3 within 1 yr; 2 in 4 yr; 1 Sudeck’s atrophy |
| Tellisi 2009 [21] | 23 | Retrospective; 5mm in OR; 16 (12-18) wk motional distraction. | AOFAS, SF-36 | 30 (12-60) | AOFAS score improved from 55 to 74 points (*P*=0.005); 91% patients reported improved pain; SF-36 scores showed modest improvement in all components. | AJDA offers a promising solution for many people with ankle arthritis. | 2 failures; failure time not reported |
| Intema 2011 [32] | 26 | Prospective; 5mm in OR; 12 to 14 wk fixed distraction. | bone density, AOS | 24 | Subchondral bone density decreased 23±12% in tibia and 18±15% in talus at 1 yr (*P*＜0.01); And still present at 2 yr. The AOS pain and disability scores showed improvement at 1 and 2 yr (*P*＜0.01). | AJDA decreased the subchondral bone density, persisted for at least 2 yr, and was correlated with clinical improvement. | no |
| Saltzman 2012 [20] | 36 | RCT; motion versus fixed distraction; 5mm in OR; 12 to 14 wk distraction. | AOS, ROM | 24 | Motion distraction group had better AOS scores than the fixed distraction group at 26, 52, and 104 weeks (*P* < 0.01). ROM reached NSD between groups. | AJDA improved the patient reported outcomes. Motion distraction showed better outcome. | 4 failures; 3 in fixed group, and 1 in motional group; 1 in 1 yr; 3 in 2 yr |
| Marijnissen 2014 [12]** | 105 | As reported by Saltzman et al [20] and Marijnissen et al [17] | Survival analysis, risk factor | 73 (24-144) | Survival analysis showed 44% of the patients failed, 17% within 2 yr and 37% within 5 yr. The percentage failure was higher in women (30% in 2 yr) than men (< 30% in 11 yr). | AJDA shows long term clinical benefit. Failure rate is considerable over the years. Females with high failure. | 37 failures; 9 in 1 yr; 9 in 2 yr; 5 in 3 yr; 5 in 4 yr; 4 in 5 yr; 5 in 6 to 17 yr |
| Nguyen 2015 [18] | 29 | As reported by Saltzman et al [20] | risk factor | 100 (60-144) | Positive predictors of ankle survival included a better AOS score at 2 years (*P*=0.04), older age (*P*=0.04), fixed distraction (*P* <0.01). | Ankle function following joint distraction declines over time. | 13 failures; 2 in 1 yr; 3 in 2 yr; 1 in 3 yr; 1 in 5 yr; 6 in 6 to 8 yr |

*FU* followup, *AOS* Ankle Osteoarthritis Score, *AOFAS* American Orthopaedic Foot and Ankle Society ankle-hindfoot scale, *SF-36* the MOS item short from health survey, *ROM* range of motion of ankle joint, *wk* weeks, *mth* months, *yr* years, *NSD* no significant difference, *AO* osteoarthritis, *AJDA* ankle joint distraction arthroplasty, *JSW* joint space width, *FWB* full weightbearing, *OR* operation room

* Two independent studies in one article reported by Marijnissen et al [16].

** Same patients reported by Saltzman et al [19] and Marijnissen et al [16] previously.
